# Development and internal validation of a predictive model for clinical deterioration in newly diagnosed lung cancer patients based on interpretable machine learning

**DOI:** 10.3389/fmed.2026.1947529

**Published:** 2026-10-07

**Authors:** Huiling Li, Yin Li, Xiaomin Huang, Zelian Zhang, Qianfei Liu, Quanfang Chen

**Affiliations:** 1 Department of Respiratory, The First Affiliated Hospital of Guangxi Medical University, Nanning, Guangxi, China; 2 Department of Pulmonary and Critical Care Medicine, The Central Hospital of Enshi Tujia and Miao Autonomous Prefecture, Enshi City, Hu Bei, China

**Keywords:** ADL score, clinical deterioration, lung cancer, machine learning, risk prediction, shap

## Abstract

**Objective:**

To develop and validate an interpretable machine learning–based model for the early identification of the risk of clinical deterioration during hospitalization in newly diagnosed lung cancer patients.

**Methods:**

Clinical data of patients with newly diagnosed lung cancer admitted between July 2023 and December 2025 were retrospectively collected in this single-center study. A hybrid feature selection strategy integrating least absolute shrinkage and selection operator (LASSO) regression and the Boruta algorithm was employed to identify key predictors. Logistic regression (LR), random forest (RF), extreme gradient boosting (XGBoost), and support vector machine (SVM) models were constructed. Model performance was evaluated using the area under the receiver operating characteristic curve (AUC-ROC), calibration curves, and decision curve analysis (DCA). SHapley Additive exPlanations (SHAP) values were further applied to interpret the model's decision-making process.

**Results:**

A total of 1,001 newly diagnosed lung cancer patients were included, comprising 121 patients (12.1%) in the deterioration group and 880 patients (87.9%) in the non-deterioration group. Through the hybrid feature selection approach, five core predictive variables were identified, including Activities of Daily Living (ADL) score, lymphocyte count, C-reactive protein (CRP), respiratory rate, and neutrophil count. Among the models, the SVM model achieved the highest observed AUC of 0.822 (95% CI: 0.737–0.899) in the internal validation set, with a sensitivity of 0.750 (95% CI: 0.594–0.893) and a specificity of 0.792 (95% CI: 0.740–0.838). After Platt scaling calibration, the model achieved a Brier score of 0.083 (Hosmer-Lemeshow *p* = 0.649), with a calibration intercept of 0.141 and a slope of 1.126, indicating satisfactory agreement between predicted and observed risks. DCA suggested a potential net clinical benefit. SHAP analysis, in conjunction with multivariable regression results, consistently demonstrated that ADL score and lymphocyte count were associated with reduced risk of clinical deterioration, whereas CRP and neutrophil count were associated with increased risk.

**Conclusion:**

The SVM model developed in this study may help identify patients at risk of clinical deterioration among hospitalized lung cancer patients and may assist healthcare professionals in achieving early risk identification and potential clinical decision support.

## 1 Introduction

Lung cancer remains the most prevalent malignancy and the leading cause of cancer-related mortality worldwide. According to the Global Cancer Statistics (GLOBOCAN) 2020, approximately 2.2 million new cases of lung cancer were diagnosed, with nearly 1.8 million deaths, ranking first among all cancer-related causes of death (1). In China, the burden of lung cancer continues to escalate, driven by population aging and environmental factors. Despite substantial advances in surgical techniques, chemotherapy, radiotherapy, as well as targeted and immunotherapeutic strategies, the safety of hospitalized lung cancer patients still faces considerable challenges (2, 3). Owing to tumor-related cachexia, impaired immune function, advanced age, and multiple comorbidities, these patients are particularly susceptible to severe clinical deterioration during hospitalization, including acute respiratory failure, severe pneumonia, and sepsis. Evidence suggests that such deterioration events not only significantly prolong hospital stay and increase healthcare costs, but also constitute major contributors to unplanned intensive care unit (ICU) admissions and in-hospital mortality (4, 5). Therefore, early and accurate risk stratification at the time of admission, with prompt identification of high-risk individuals, is of critical clinical importance for optimizing decision-making and implementing timely preventive interventions.

Currently, early warning scoring systems widely used in clinical practice, such as the National Early Warning Score 2 (NEWS2) and Modified Early Warning Score (MEWS), primarily rely on routine vital signs including respiratory rate, heart rate, and blood pressure. However, these tools were originally developed for general medical and surgical ward populations and exhibit notable limitations when applied to patients with lung cancer. The clinical course of lung cancer is highly heterogeneous, and the risk of deterioration is influenced not only by fluctuations in vital signs but also by factors such as functional reserve, tumor burden, and systemic inflammatory status. Conventional scoring systems often fail to capture these deeper risk signals, resulting in suboptimal predictive sensitivity (6, 7). The Activities of Daily Living (ADL) score, as a core indicator of baseline functional status, has been extensively validated in geriatric medicine and rehabilitation settings. ADL not only reflects a patient's physiological capacity but also indirectly indicates their ability to compensate for acute stress (8). Previous studies have demonstrated a strong association between impaired ADL at admission and an increased risk of mortality in patients with malignancies (9). In newly diagnosed lung cancer patients, ADL impairment often signifies a higher risk of frailty, suggesting an increased susceptibility to organ dysfunction during clinical interventions (10). Nevertheless, reliance on ADL alone is insufficient to capture the complexity of underlying pathophysiological changes. Therefore, the integration of functional status indicators with peripheral blood inflammatory markers and biochemical parameters to construct a comprehensive assessment model remains a critical challenge in the clinical management and nursing care of lung cancer patients.

Machine learning (ML) has emerged as a powerful approach to address this clinical challenge. Compared with traditional statistical models, ML algorithms—such as random forest (RF) and extreme gradient boosting (XGBoost)—are capable of capturing complex nonlinear relationships among variables, handling high-dimensional datasets, and achieving superior predictive performance in heterogeneous populations (11). To date, ML has demonstrated excellent performance in areas such as sepsis early warning and acute kidney injury prediction; however, its application in predicting in-hospital deterioration in lung cancer patients based on ADL scores combined with clinical indicators remains in its early stages (12). Therefore, this study aims to develop an early and rapid warning model for clinical deterioration during hospitalization in lung cancer patients by integrating admission ADL scores with routine laboratory parameters, including complete blood count and blood gas analysis, using multiple ML algorithms. This approach will enable more precise management of hospitalized lung cancer patients. It is intended for use by the clinical team-including physicians, nurse practitioners-to identify high-risk patients who may benefit from intensified monitoring, early multidisciplinary consultation, or proactive escalation of care.

## 2 Methods

### 2.1 Study population

In this retrospective study, clinical data were collected from patients who were newly diagnosed with lung cancer and admitted to the Department of Respiratory and Critical Care Medicine between July 2023 and December 2025. The inclusion criteria were as follows: (1) primary lung cancer confirmed by histopathological or cytological examination; and (2) age ≥18 years. The exclusion criteria included: (1) missing ADL data (*n* = 215); and (2) concomitant presence of other malignancies. A total of 1,001 patients were ultimately enrolled. The primary outcome was clinical deterioration during hospitalization, defined as the occurrence of any of the following events: (1) transfer to the intensive care unit (ICU); (2) requirement for mechanical ventilation; (3) in-hospital death. This retrospective study complied with the Declaration of Helsinki and was approved by the Ethics Committee of Enshi Autonomous Prefecture Central Hospital (Approval No. 2026-070-01) on May 14, 2026, which granted a waiver of informed consent.

### 2.2 Data collection

Clinical data were extracted from the hospital electronic medical record system, including demographic characteristics, vital signs, and laboratory parameters. All predictor values were the first measurements recorded after admission. (1) Demographic characteristics: sex and age; (2) ADL score at admission: assessed using the Barthel Index, with a total score ranging from 0 to 100; (3) vital signs: body temperature, pulse rate, respiratory rate, systolic blood pressure, and diastolic blood pressure; (4) personal history: smoking history and alcohol consumption history; (5) comorbidities: hypertension, diabetes mellitus, chronic lung disease, and coronary artery disease; (6) laboratory parameters: complete blood count [white blood cells, red blood cells, hemoglobin, platelets, lymphocytes, neutrophils, monocytes, and red cell distribution width–coefficient of variation (RDW-CV)], C-reactive protein (CRP), and arterial blood gas analysis [pH, partial pressure of oxygen (PaO_2_), partial pressure of carbon dioxide (PaCO_2_), oxygen saturation (SO_2_), lactate (Lac), sodium, potassium, chloride, calcium, bicarbonate, base excess (BE), and oxygenation index]. NEWS2 and MEWS scores were calculated from the admission vital signs, including respiratory rate, oxygen saturation, pulse rate, systolic blood pressure, and body temperature. Because supplemental oxygen use and level of consciousness were not recorded in the electronic medical records, these two components could not be scored, and the resulting scores were slightly conservative. Both scores were used only as comparators against the machine learning model and were not entered into the feature selection procedure.

### 2.3 Hybrid feature selection strategy

In this study, the entire sample was randomly split into a training set and a validation set at a 7:3 ratio, and all feature selection procedures were strictly confined to the training set. To enhance predictive performance while reducing multicollinearity and the risk of overfitting, a hybrid feature selection strategy integrating both linear and nonlinear algorithms was employed. All preprocessing steps—including missing value imputation, standardization, and feature selection—as well as subsequent model training, hyperparameter tuning, class weighting, probability calibration, and threshold determination were performed exclusively on the training set; the validation set was used solely for independent evaluation of the final models.

First, least absolute shrinkage and selection operator (LASSO) regression was applied to eliminate irrelevant variables through coefficient penalization and shrinkage. The optimal regularization parameter (*λ*) was selected via 10-fold cross-validation using the lambda.min criterion (i.e., the *λ* value that minimized the cross-validated deviance). Simultaneously, the Boruta algorithm, based on RF, was utilized to identify features with global importance for the outcome variable. The algorithm was configured with maxRuns = 100, a significance threshold of *p* < 0.05, and random_state = 42 to ensure reproducibility. Feature importance rankings were evaluated across multiple iterations to confirm stability of the selection results. The intersection of features selected by both methods was then used as the final set of predictors for subsequent ML modeling, thereby ensuring both clinical relevance and statistical robustness of the selected variables.

### 2.4 Model construction and performance evaluation

In this study, four widely used ML models were developed to predict the risk of clinical deterioration, including logistic regression (LR), random forest (RF), extreme gradient boosting (XGBoost), and support vector machine (SVM). All models were trained and evaluated exclusively within the training set framework; the independent validation set was used solely for final performance assessment. To address class imbalance, class weighting was applied with weights inversely proportional to class frequencies. Specifically, LR was trained with balanced class weights, RF with 200 trees and a maximum depth of 5, SVM with a radial basis function kernel, and XGBoost with 100 trees and a maximum depth of 3, with the scale_pos_weight parameter of XGBoost set to the ratio of non-events to events. All models used a fixed random seed of 42. The discriminative ability of each model was quantified and compared using the area under the receiver operating characteristic curve (AUC-ROC). The optimal diagnostic cut-off point was determined by maximizing the Youden index on the training set, and this fixed threshold was subsequently applied to the validation set. The final predictive model was selected by comparing sensitivity, specificity, accuracy, and the F1 score across the candidate models, with priority given to sensitivity in view of the intended admission-screening use. Model performance was further validated using bootstrap resampling (1,000 iterations) to estimate 95% confidence intervals for all performance metrics.

### 2.5 Model calibration and clinical utility assessment

All model calibration and clinical utility assessments were performed on the independent validation set. Probability calibration was performed using Platt scaling (sigmoid method) with 5-fold cross-validation on the training set to avoid data leakage. Calibration curves were plotted to assess the agreement between predicted probabilities and observed event rates. To further evaluate the clinical applicability of the model, decision curve analysis (DCA) was conducted by comparing the net benefit of using the model to guide intervention strategies against the “treat-all” and “treat-none” approaches across a range of threshold probabilities. Model calibration was further evaluated using the Brier score, calibration intercept, calibration slope, and the Hosmer–Lemeshow goodness-of-fit test, with 95% confidence intervals for the calibration intercept and slope estimated by bootstrap resampling.

### 2.6 Model interpretability and multivariable analysis validation

To address the “black-box” nature of ML models, SHapley Additive exPlanations (SHAP) were introduced to enhance interpretability. By quantifying both global feature importance and individual feature contributions, SHAP enables a detailed assessment of the impact of each predictor on the final model output. To examine the independent association of the selected features with clinical deterioration, a separate multivariable LR model was constructed using the five core variables on the training set. This step aimed to assess the independent association of each feature with clinical deterioration, adjusting for potential confounding. The results were presented as adjusted odds ratios (ORs), 95% confidence intervals (CIs), and corresponding *P* values.

### 2.7 Statistical analysis

All data processing and statistical analyses were conducted using Python (version 3.13.5). For baseline characteristics, continuous variables were tested for normality and found to follow non-normal distributions; therefore, they were described using the median and interquartile range [median (Q1, Q3)], and comparisons between groups were performed using the nonparametric Mann–Whitney *U* test. Categorical variables were expressed as frequencies and percentages (*n*, %), and comparisons were conducted using the chi-square test or Fisher's exact test, as appropriate. All statistical tests were two-sided, with *p* < 0.05 considered statistically significant. After splitting data into training and validation sets (7:3 ratio) via stratified random sampling, missing data were imputed using iterative chained-equation imputation (IterativeImputer in scikit-learn), applied exclusively to the training set to prevent data leakage. To address the common issue of class imbalance in medical datasets—where the number of deterioration cases is smaller than that of non-deterioration cases—cost-sensitive learning was incorporated during model training by assigning higher weights to the minority class, thereby mitigating potential bias in model learning. The main Python libraries included scikit-learn (version 1.9.0), XGBoost (3.2.0), SHAP (0.51.0), and statsmodels (0.14.6).

## 3 Results

### 3.1 Baseline characteristics and univariate analysis

A total of 1,001 patients were included in this study (Figure 1), comprising 880 patients without clinical deterioration and 121 patients with clinical deterioration (Table 1). Univariate analysis revealed that, compared with the non-deterioration group, patients in the deterioration group were significantly older (*p* = 0.016) and had a markedly higher prevalence of coronary artery disease (CAD) (10.7% vs. 2.6%, *p* < 0.001). In terms of admission vital signs and clinical assessment, the deterioration group exhibited significantly lower ADL scores than the non-deterioration group (median 65.0 vs. 100.0, *p* < 0.001), along with significantly elevated pulse rate and respiratory rate (both *p* < 0.001). Regarding laboratory findings, inflammatory markers in the deterioration group, including CRP, white blood cell count (WBC), and neutrophil count, were significantly increased, whereas lymphocyte count was markedly decreased. In addition, hemoglobin, red blood cell count, and arterial blood gas analysis (Ca, Cl, and Na) were significantly lower in the deterioration group, while lactate (Lac) levels were significantly higher (all *p* < 0.001). NEWS2 and MEWS scores were also significantly higher in the deterioration group than in the non-deterioration group (both *p* < 0.001; Table 1).

**Figure 1:**
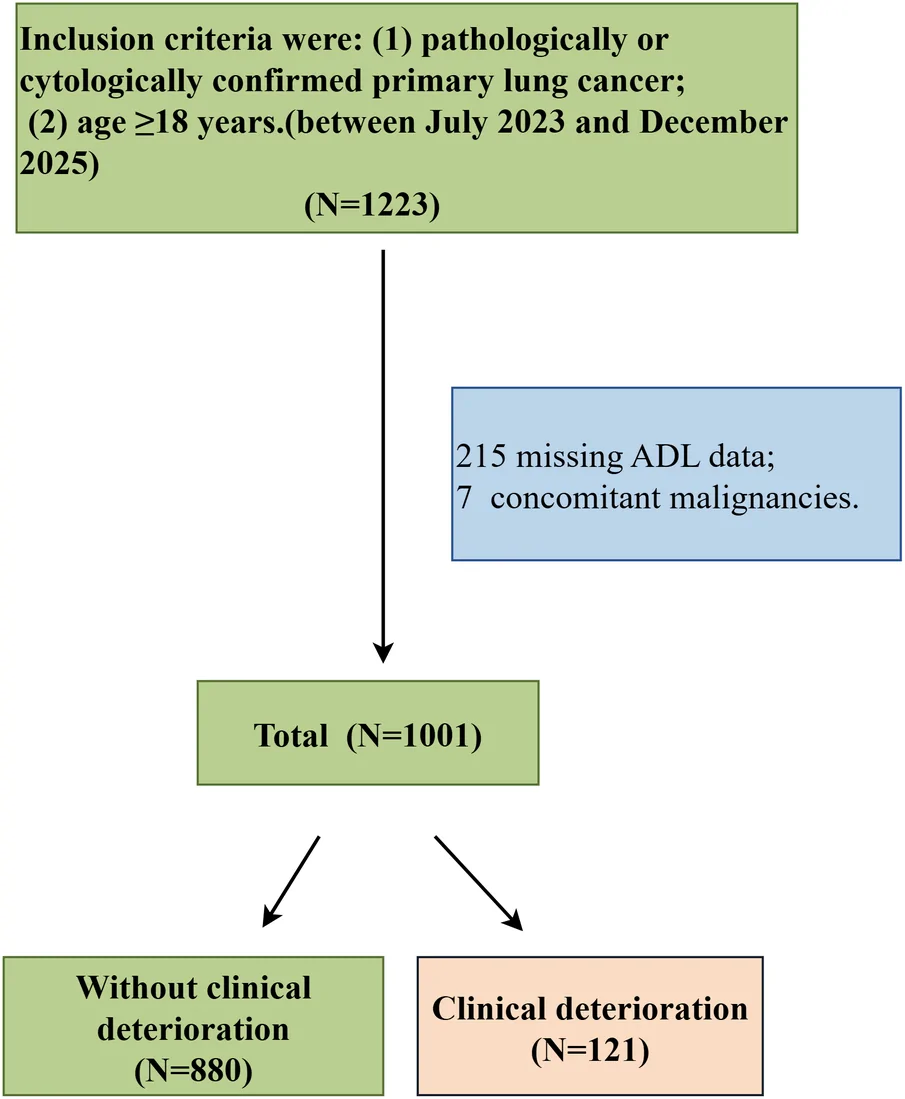
Flowchart of study population enrollment and exclusion criteria.

**Table 1:** Baseline characteristics of the study population stratified by clinical deterioration status.

Characteristics	Overall (*N* = 1,001)	Without clinical deterioration (*N* = 880)	Clinical deterioration (*N* = 121)	*P*-Value
Gender(Male), *n* (%)	695 (69.4)	606 (68.9)	89 (73.6)	0.345
Smoking, *n* (%)	623 (62.2)	550 (62.5)	73 (60.3)	0.718
Drinking, *n* (%)	307 (30.7)	275 (31.2)	32 (26.4)	0.332
HBP, *n* (%)	144 (14.4)	124 (14.1)	20 (16.5)	0.563
DM, *n* (%)	65 (6.5)	60 (6.8)	5 (4.1)	0.354
CLD, *n* (%)	179 (17.9)	154 (17.5)	25 (20.7)	0.469
CAD, *n* (%)	36 (3.6)	23 (2.6)	13 (10.7)	<0.001
Age, median [Q1, Q3]	66.0 [58.0, 72.0]	66.0 [58.0, 72.0]	68.0 [60.0, 73.0]	0.016
ADL, median [Q1, Q3]	100.0 [90.0, 100.0]	100.0 [90.0, 100.0]	65.0 [50.0, 95.0]	<0.001
Temperature, median [Q1, Q3]	36.5 [36.5, 36.6]	36.5 [36.5, 36.6]	36.5 [36.4, 36.6]	0.943
Pulse, median [Q1, Q3]	80.0 [77.0, 89.0]	80.0 [76.0, 88.0]	89.0 [79.0, 106.0]	<0.001
Resp_Rate, median [Q1, Q3]	20.0 [19.0, 21.0]	20.0 [19.0, 20.0]	20.0 [19.0, 24.0]	<0.001
SBP, median [Q1, Q3]	126.0 [120.0, 133.5]	126.0 [120.0, 133.0]	126.0 [118.0, 139.0]	0.398
DBP, median [Q1, Q3]	78.0 [70.0, 84.0]	78.0 [70.0, 83.0]	77.0 [70.0, 86.0]	0.990
BE(B), median [Q1, Q3]	0.3 [−1.0, 1.7]	0.3 [−1.0, 1.6]	0.3 [−1.6, 2.2]	0.794
CRP, median [Q1, Q3]	10.3 [2.7, 47.4]	8.8 [2.5, 34.4]	54.6 [10.7, 114.1]	<0.001
Ca, median [Q1, Q3]	1.1 [1.1, 1.2]	1.1 [1.1, 1.2]	1.1 [1.1, 1.2]	<0.001
Cl, median [Q1, Q3]	107.3 [105.0, 109.3]	107.7 [105.0, 109.6]	105.0 [101.8, 108.1]	<0.001
HCO3, median [Q1, Q3]	24.6 [23.0, 26.1]	24.6 [23.1, 26.1]	24.5 [22.1, 27.1]	0.687
HGB, median [Q1, Q3]	122.0 [109.0, 133.0]	122.0 [110.0, 133.0]	115.0 [100.5, 129.0]	<0.001
K, median [Q1, Q3]	3.7 [3.5, 3.9]	3.7 [3.5, 3.9]	3.7 [3.4, 4.0]	0.276
Lymphocyte, median [Q1, Q3]	1.2 [0.8, 1.6]	1.2 [0.9, 1.6]	0.7 [0.5, 1.1]	<0.001
Lac, median [Q1, Q3]	1.3 [1.0, 1.7]	1.3 [1.0, 1.7]	1.6 [1.2, 2.1]	<0.001
Monocyte, median [Q1, Q3]	0.4 [0.3, 0.6]	0.4 [0.3, 0.6]	0.5 [0.3, 0.7]	0.054
Neutrophil, median [Q1, Q3]	4.4 [3.2, 5.9]	4.2 [3.1, 5.6]	5.9 [4.1, 9.4]	<0.001
Na, median [Q1, Q3]	139.0 [136.3, 141.0]	139.0 [136.8, 141.0]	137.4 [134.6, 140.7]	<0.001
PCO2, median [Q1, Q3]	37.9 [35.0, 40.6]	38.0 [35.3, 40.4]	37.4 [33.0, 42.5]	0.571
PF Index, median [Q1, Q3]	355.5 [317.5, 390.7]	357.0 [319.2, 390.4]	343.8 [289.5, 393.2]	0.117
PH, median [Q1, Q3]	7.4 [7.4, 7.4]	7.4 [7.4, 7.4]	7.4 [7.4, 7.5]	0.662
PLT, median [Q1, Q3]	213.0 [170.0, 273.0]	213.0 [170.2, 272.8]	215.0 [163.5, 278.0]	0.677
PO2, median [Q1,Q3]	74.9 [67.3, 82.2]	75.1 [67.5, 82.0]	73.9 [62.0, 87.2]	0.854
RBC, median [Q1, Q3]	4.0 [3.7, 4.4]	4.1 [3.7, 4.4]	3.9 [3.4, 4.4]	0.006
RDW-CV, median [Q1, Q3]	13.3 [12.8, 14.1]	13.2 [12.7, 14.1]	13.9 [13.1, 15.1]	<0.001
SO2, median [Q1, Q3]	96.1 [94.3, 97.4]	96.1 [94.4, 97.4]	95.9 [92.5, 98.0]	0.312
WBC, median [Q1, Q3]	6.3 [4.9, 8.1]	6.1 [4.8, 7.8]	7.5 [5.7, 10.8]	<0.001
NEWS2, median [Q1, Q3]	2.0 [1.0, 3.0]	1.0 [1.0, 3.0]	3.0 [1.0, 5.0]	<0.001
MEWS, median [Q1, Q3]	0.0 [0.0, 1.0]	0.0 [0.0, 1.0]	1.0 [0.0, 2.0]	<0.001
Clinical deterioration, *n* (%)	121 (12.1)	—	—	—
ICU transfer, *n* (%)	98 (9.8)	0 (0.0)	98 (81.0)	—
Mechanical ventilation, *n* (%)	40 (4.0)	0 (0.0)	40 (33.1)	—
In-hospital death, *n* (%)	23 (2.3)	0 (0.0)	23 (19.0)	—

ADL, activities of daily living; BE, base excess; CAD, coronary artery disease; CLD, chronic lung disease; CRP, C-reactive protein; DBP, diastolic blood pressure; DM, diabetes mellitus; HBP, hypertension; HCO₃, bicarbonate; HGB, hemoglobin; Lac, lactate; PaCO_2_, partial pressure of carbon dioxide; PaO_2_, partial pressure of oxygen; PF Index, oxygenation index (PaO_2_/FiO_2_ ratio); PLT, platelets; RBC, red blood cells; RDW-CV, red cell distribution width–coefficient of variation; SBP, systolic blood pressure; SO_2_, oxygen saturation; WBC, white blood cells; NEWS2, National Early Warning Score 2; MEWS, Modified Early Warning Score.

Continuous variables are expressed as median [Q1, Q3]; categorical variables as *n* (%).

### 3.2 Results of hybrid feature selection

To develop an efficient predictive model, a hybrid feature selection approach was applied to the candidate variables (Figure 2). LASSO regression retained five features with non-zero coefficients at the optimal *λ* value (Figure 2A), while the RF–based Boruta algorithm identified 11 features as highly important (Figure 2B). By taking the intersection of the results from both methods (Venn diagram, Figure 2C), five core predictive variables were ultimately identified: ADL score, respiratory rate (Respiratory Rate), CRP, lymphocyte count, and neutrophil count (Neutrophil). Because the two algorithms rely on different selection principles, the intersection retained only variables supported by both linear shrinkage and nonlinear importance-based selection. Bootstrap resampling (200 iterations) confirmed the stability of feature selection, with ADL, CRP, and lymphocyte count selected in more than 90% of the resamples (Figure 2D). Moreover, all variables can be obtained within 30 min of admission through bedside assessment or point-of-care testing (POCT), and were therefore retained for subsequent model development.

**Figure 2:**
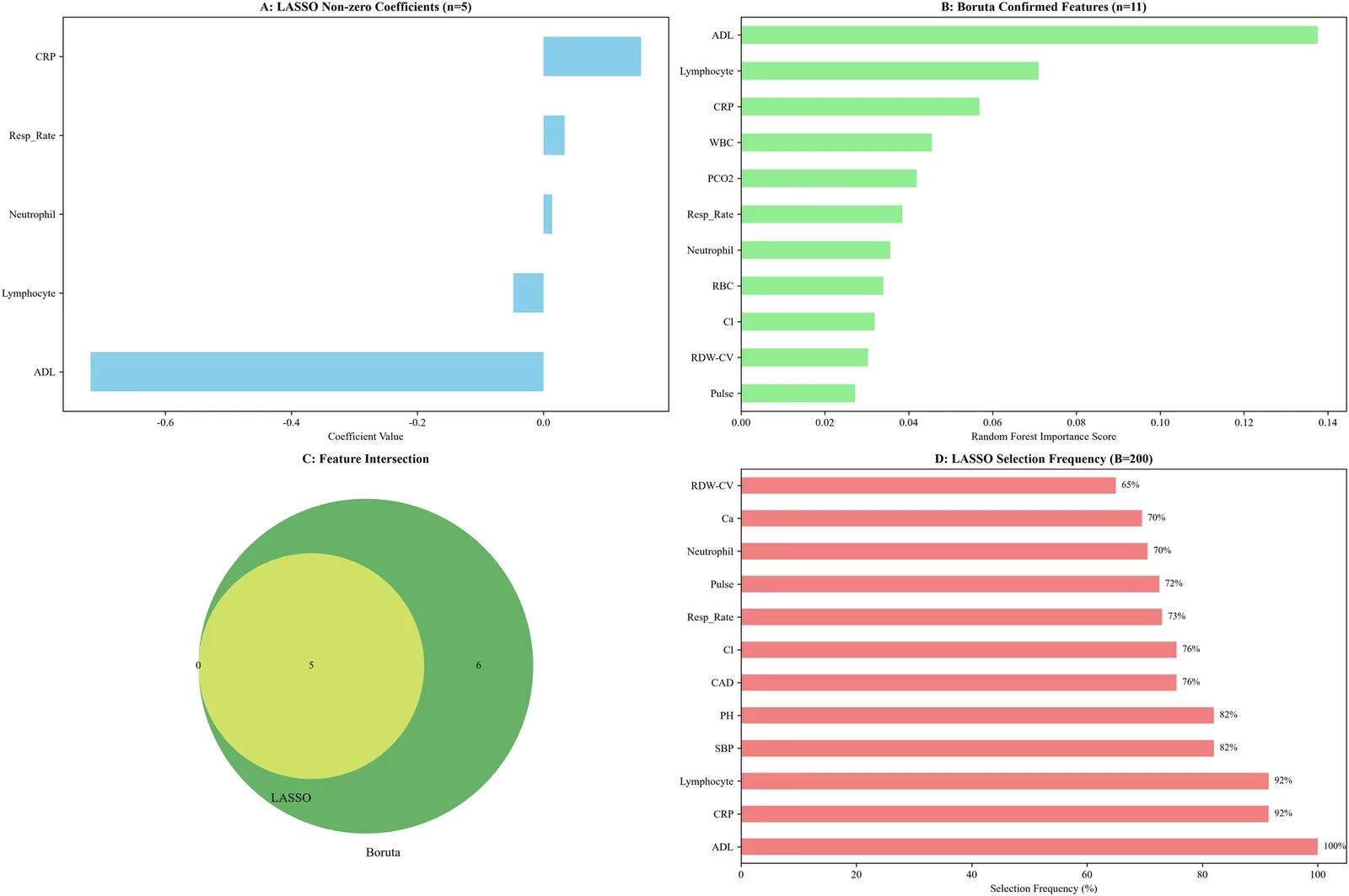
Hybrid feature selection process using LASSO and boruta algorithms. **(A)** LASSO coefficient profiles of candidate predictors. **(B)** Boruta algorithm-based feature importance ranking. **(C)** Venn diagram showing the intersection of features selected by both methods. **(D)** Bootstrap selection frequency of each candidate predictor across 200 resamples.

### 3.3 Comparison of ML model performance

After stratified random splitting, the training set comprised 700 patients with 85 events (12.1%), and the validation set comprised 301 patients with 36 events (12.0%). Using the five selected core features, four ML models were trained and evaluated (Figure 3). ROC curve analysis (Figure 3A) and the comprehensive performance heatmap (Figure 3B) indicated that the SVM model achieved the highest observed AUC in the validation set (0.822, 95% CI: 0.737–0.899), followed by LR (0.811, 95% CI: 0.692–0.902), RF (0.795, 95% CI: 0.689–0.884), and XGBoost (0.782, 95% CI: 0.680–0.871). SVM also demonstrated the highest sensitivity (0.750, 95% CI: 0.594–0.893) among all models, while maintaining a specificity of 0.792 (95% CI: 0.740–0.838) and an accuracy of 0.787 (95% CI: 0.738–0.831). In admission screening for clinical deterioration, minimizing false negatives is clinically prioritized over reducing false positives. Although the AUC differences among the four models were modest, SVM was not regarded as statistically superior to the other algorithms. It was retained as the final predictive model for the clinical reason just stated, namely its highest sensitivity at the fixed threshold. The comprehensive performance metrics of all four models are presented in Table 2.

**Figure 3:**
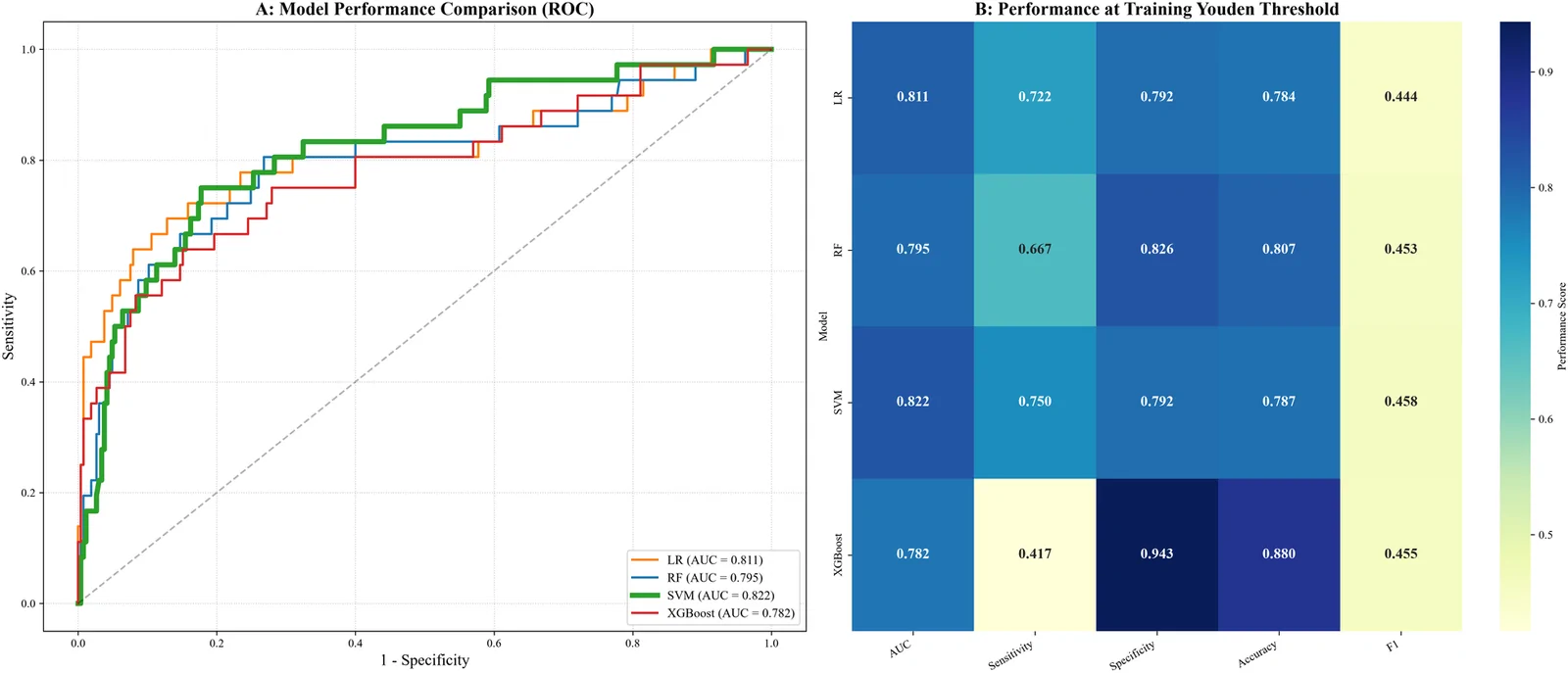
Performance comparison of machine learning models in the validation cohort. **(A)** Model Performance Comparison (ROC). **(B)** Comprehensive Performance Heatmap.

**Table 2:** Performance metrics of machine learning models with 95% confidence intervals in the internal validation set.

Model	AUC (95% CI)	Sensitivity (95% CI)	Specificity (95% CI)	F1-score (95% CI)	Accuracy (95% CI)
LR	0.811 (0.692–0.902)	0.722 (0.556–0.865)	0.792 (0.740–0.841)	0.444 (0.321–0.553)	0.784 (0.734–0.831)
RF	0.795 (0.689–0.884)	0.667 (0.500–0.824)	0.826 (0.777–0.872)	0.453 (0.323–0.563)	0.807 (0.761–0.850)
SVM	0.822 (0.737–0.899)	0.750 (0.594–0.893)	0.792 (0.740–0.838)	0.458 (0.337–0.569)	0.787 (0.738–0.831)
XGBoost	0.782 (0.680–0.871)	0.417 (0.257–0.581)	0.943 (0.914–0.970)	0.455 (0.294–0.597)	0.880 (0.847–0.914)

CI, confidence interval; LR, logistic regression; RF, random forest; XGBoost, extreme gradient boosting; SVM, support vector machine.

95% confidence intervals were estimated using bootstrap resampling (1,000 iterations) at a fixed threshold. For each model, the optimal cut-off was determined by maximizing the Youden index on the training set and was then applied to the validation set. The fixed cut-offs for LR, RF, SVM, and XGBoost were 0.467, 0.430, 0.107, and 0.763, respectively. AUC confidence intervals were calculated using DeLong's method.

### 3.4 Stability and clinical reliability of the final model

For the final selected SVM model, we assessed its predictive stability and clinical reliability in the validation set (Figure 4). After Platt scaling calibration with 5-fold cross-validation on the training set, the calibrated SVM model achieved an AUC of 0.816 (95% CI: 0.724–0.894) in the validation set, compared with 0.822 before calibration (Table 2); the small difference reflects the internal cross-validation procedure and does not indicate a change in discriminative ability. ROC analysis showed limited evidence of overfitting (training AUC vs. validation AUC, Figure 4A). The optimal probability cut-off was determined by maximizing the Youden index on the training set (0.102), and this fixed threshold was then applied to the validation set. The calibration curve showed close agreement between predicted and observed event rates (Figure 4B), with a calibration intercept of 0.141 (95% CI: −0.514 to 0.820) and a slope of 1.126 (95% CI: 0.808–1.511) (Table 3). The Hosmer–Lemeshow test yielded a *χ*^2^ of 5.988 (*p* = 0.649); given the limited number of events, this result should be interpreted with caution. The Brier score was 0.083. The model achieved a sensitivity of 0.750 (95% CI: 0.594–0.893), a specificity of 0.777 (95% CI: 0.725–0.825), a PPV of 0.314, and an NPV of 0.958 in the validation set. DCA (Figure 4C) confirmed that across a range of clinically relevant threshold probabilities, the net clinical benefit of the SVM model was consistently superior to the treat-all or treat-none strategies. For comparison, NEWS2 and MEWS achieved validation AUCs of 0.639 (95% CI: 0.527–0.732) and 0.609 (95% CI: 0.514–0.704), respectively, both lower than the SVM model (Figures 5A,B). The comprehensive performance metrics of the calibrated model are summarized in Table 3.

**Figure 4:**
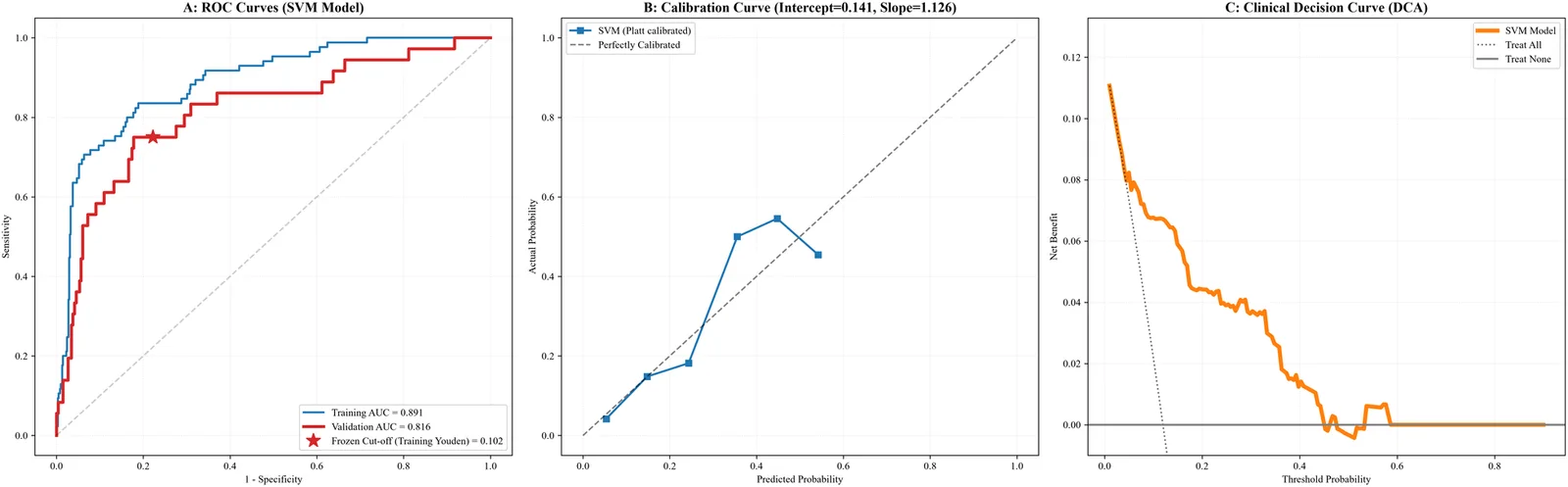
Performance evaluation of the final SVM model. **(A)** ROC curves of the final SVM model in training and validation cohorts. **(B)** Calibration curve comparing predicted probabilities and observed outcomes. **(C)** Decision curve analysis (DCA) demonstrating the net clinical benefit across threshold probabilities.

**Table 3:** Detailed performance metrics of the final calibrated SVM model in the internal validation set.

Metric	Value (95% CI)
Discrimination	
AUC	0.816 (0.724–0.894)
Threshold	
Optimal cut-off (Youden index)	0.102
Classification performance	
Accuracy	0.774 (0.721–0.824)
Sensitivity	0.750 (0.594–0.893)
Specificity	0.777 (0.725–0.825)
Positive predictive value (PPV)	0.314 (0.218–0.418)
Negative predictive value (NPV)	0.958 (0.929–0.985)
F1-score	0.443 (0.325–0.550)
Calibration	
Brier score	0.083 (0.063–0.107)
Calibration intercept	0.141 (−0.514 to 0.820)
Calibration slope	1.126 (0.808 to 1.511)
Hosmer-Lemeshow *χ*^2^	5.988
Hosmer-Lemeshow *p*-value	0.649

AUC, area under the receiver operating characteristic curve; PPV, positive predictive value; NPV, negative predictive value. 95% confidence intervals were estimated using bootstrap resampling (1,000 iterations). The optimal cut-off was determined by maximizing the Youden index on the training set and applied to the validation set. Calibration was assessed using the Brier score, calibration intercept/slope, and the Hosmer-Lemeshow goodness-of-fit test.

**Figure 5:**
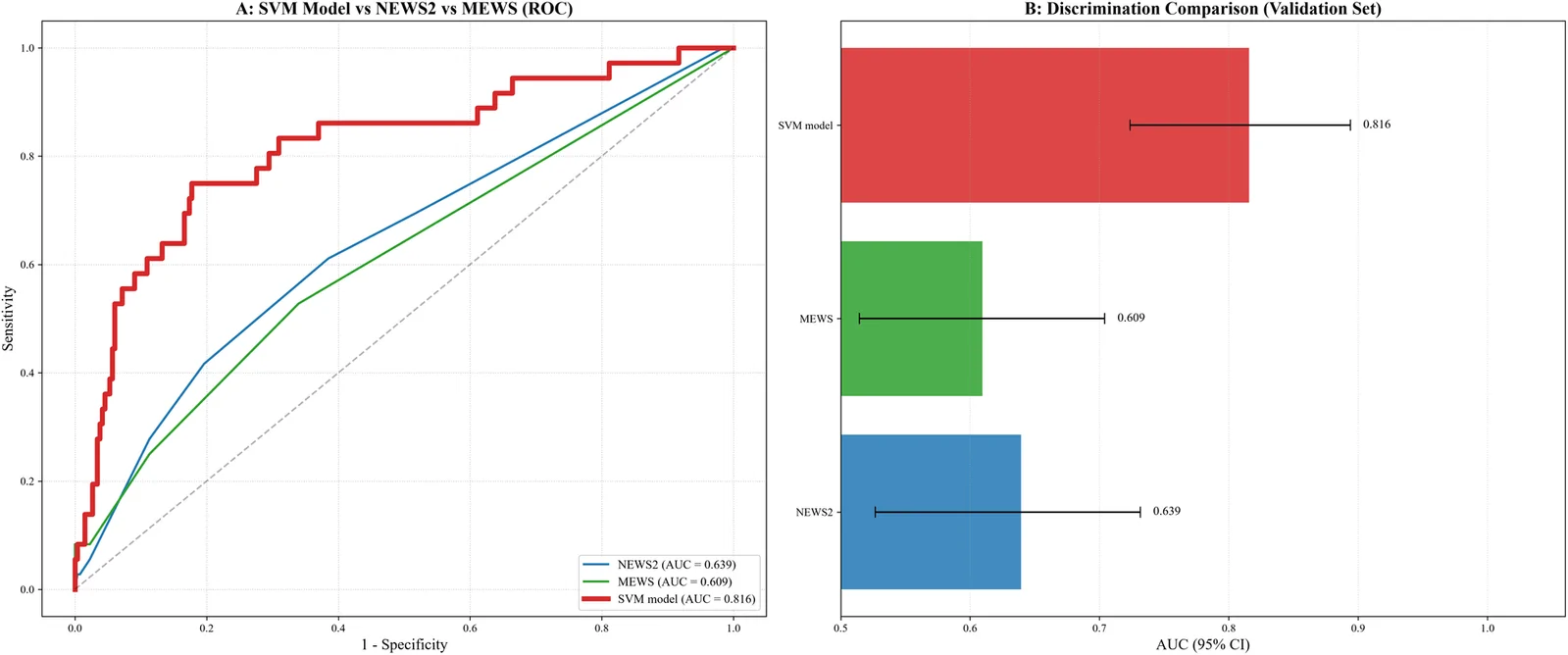
Comparison of the SVM model with NEWS2 and MEWS in the validation set. **(A)** ROC curves of the SVM model, NEWS2, and MEWS. **(B)** AUC values with 95% confidence intervals.

### 3.5 SHAP-based feature importance and model interpretability

To elucidate the internal decision-making mechanism of the SVM model, SHAP analysis was performed using KernelExplainer (Figure 6). The combination of the SHAP summary plot (Figure 6A) and the global feature importance ranking (Figure 6B) revealed that ADL score, CRP, and neutrophil count were the top three driving factors of the prediction model, with mean absolute SHAP values of 0.06, 0.02, and 0.02, respectively. Lower ADL scores and lymphocyte depletion (blue dots distributed on the right side with positive SHAP values) were associated with a markedly increased predicted risk of deterioration. Meanwhile, elevated CRP levels, increased neutrophil counts, and higher respiratory rates (red dots located on the right side) were also associated with a higher predicted risk of deterioration. Furthermore, the SHAP decision plot (Figure 6C) dynamically illustrated how the model output for individual patients in the validation set was progressively shifted from baseline toward higher or lower risk regions through the cumulative effects of multiple features.

**Figure 6:**
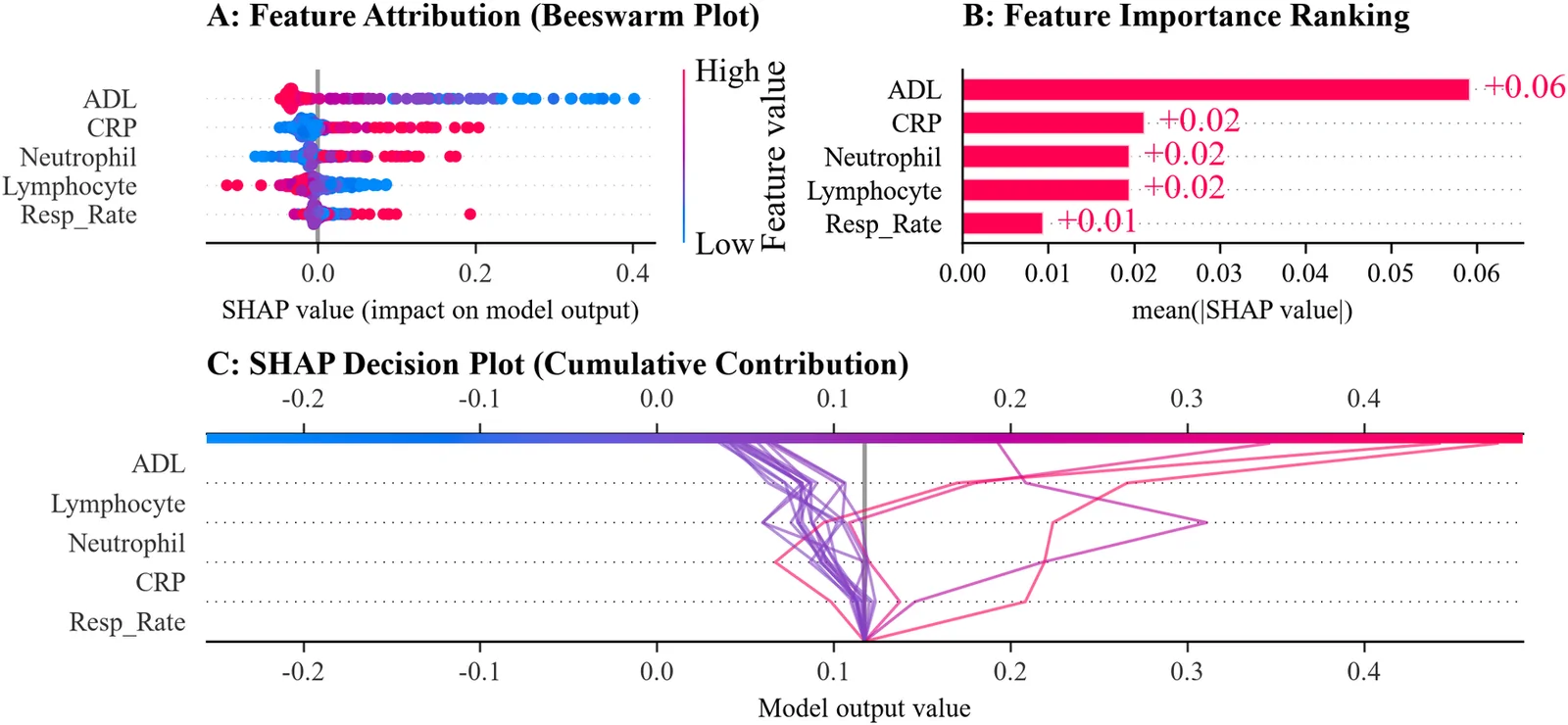
SHAP analysis for model interpretability. **(A)** Beeswarm plot of SHAP values for top predictors. **(B)** Global feature importance ranking based on mean absolute SHAP values. **(C)** Individualized decision plot illustrating cumulative contributions of features to final risk predictions.

### 3.6 Multivariable statistical analysis of SHAP-derived features

To further examine the independent association of the features selected by the ML model with clinical deterioration, the five core variables were incorporated into a multivariable LR model based on the training set. As shown in Table 4, a higher ADL score (OR = 0.951, 95% CI: 0.936–0.966, *p* < 0.001) and lymphocyte count (OR = 0.325, 95% CI: 0.169–0.623, *p* < 0.001) were identified as factors associated with reduced risk against clinical deterioration. In contrast, CRP (OR = 1.005, 95% CI: 1.001–1.010, *P* = 0.024) and neutrophil count (OR = 1.086, 95% CI: 1.003–1.175, *p* = 0.042) were confirmed as independent risk factors associated with increased likelihood of deterioration. Interestingly, although respiratory rate (Respiratory Rate, *p* = 0.103) did not reach statistical significance in the conventional multivariable linear model, it was assigned high importance in the SHAP analysis.

**Table 4:** Multivariate logistic regression analysis of predictors for clinical deterioration.

Variable	*β* (Coefficient)	OR (95% CI)	*P*-Value
ADL	−0.0505	0.951 (0.936–0.966)	<0.001
Lymphocyte	−1.1253	0.325 (0.169–0.623)	<0.001
CRP	0.0053	1.005 (1.001–1.010)	0.024
Resp_Rate	0.0936	1.098 (0.981–1.229)	0.103
Neutrophil	0.0821	1.086 (1.003–1.175)	0.042

ADL, Activities of Daily Living; CRP, C-reactive protein; OR, odds ratio; CI, confidence interval.

## 4 Discussion

In this study, through a retrospective analysis of clinical data from 1,001 patients and the integration of a hybrid feature selection strategy combining LASSO and Boruta algorithms, we developed and internally evaluated a multidimensional early warning model. The results demonstrated that among multiple ML algorithms, the SVM model showed the highest observed discrimination (AUC = 0.822 and sensitivity = 0.750 in the validation set), along with generally acceptable discriminative ability and satisfactory calibration (calibration intercept = 0.141, slope = 1.126; Hosmer–Lemeshow *p* = 0.649; Brier score = 0.083) and a favorable net clinical benefit on DCA. More importantly, by incorporating SHAP-based interpretability analysis and cross-validating the findings with multivariable regression, we identified baseline ADL score, CRP, and neutrophil count as the most critical predictors associated with the risk of clinical deterioration. These findings highlight the key clinical characteristics correlated with early deterioration in lung cancer patients and may provide an objective basis for individualized risk stratification and optimized allocation of medical resources at the time of hospital admission.

Within the multidimensional indicator framework, the ADL score emerged as the variable most strongly associated with the prediction of clinical deterioration in lung cancer patients. In the present study, patients in the deterioration group had significantly lower ADL scores compared with those in the non-deterioration group (median 65.0 vs. 100.0, *p* < 0.001). The prominent predictive weight of the ADL score may reflect its comprehensive reflection of the patient's systemic vulnerability. Vermeiren et al. (13) proposed that frailty is a complex geriatric syndrome characterized by diminished physiological reserve across multiple systems, rendering individuals more susceptible to stressors and significantly impairing their ability to perform ADL (14). Furthermore, kinematic studies by Schmidle et al. (15) demonstrated that impairments in ADL performance among frail individuals primarily stem from underlying physiological deficits rather than isolated declines in cognitive or sensorimotor control. This suggests that a low ADL score is indicative of depleted physiological reserve, predisposing patients to rapid decompensation and cascading organ dysfunction even in response to relatively mild in-hospital insults, such as minor infections or early respiratory compromise.

In oncology-specific populations, the decline in ADL carries even more profound clinical implications. A meta-analysis involving adult cancer patients (16) reported that ADL-related disability is highly prevalent among hospitalized individuals (with a prevalence of 54.6%), with fundamental activities such as ambulation and transfer being most commonly affected. In patients with lung cancer, such functional limitations may directly contribute to ventilation–perfusion mismatch and impaired airway clearance, thereby significantly increasing the risk of microatelectasis and hospital-acquired complications. Moreover, a study focusing on patients with advanced cancer and chronic obstructive pulmonary disease demonstrated that loss of basic ADL function is independently associated with an increased symptom burden (17). These findings help to explain why lung cancer patients with lower ADL scores in this study were more likely to experience rapid clinical deterioration during hospitalization.

Beyond functional status, the present model further highlights the potential predictive value of peripheral blood biomarkers, including neutrophil count, lymphocyte count, and CRP, in identifying clinical deterioration. The dynamic alterations of these routine laboratory indicators collectively reflect a dysregulated immune–inflammatory microenvironment within the host (18). Previous studies have demonstrated that inflammation is deeply involved in multiple stages of tumor development, including malignant transformation, invasion, and metastasis (19, 20). In patients with lung cancer, tumor-induced systemic inflammation represents one of the most characteristic interactions between the host and the tumor. A heightened inflammatory burden not only depletes the body's physiological reserves (21), but is also manifested at the cellular level by marked elevations in peripheral neutrophil counts and CRP levels, accompanied by significant lymphocyte depletion. A reduction in lymphocytes directly impairs antitumor immune surveillance and clearance. In contrast, an abnormal increase in neutrophils is often associated with the release of large amounts of pro-inflammatory mediators, further reshaping an immunosuppressive microenvironment and thereby accelerating disease progression or acute clinical deterioration (22–25).

The Inflammatory Burden Index (IBI), proposed by the team of Hailun Xie, defined as CRP × neutrophil count/lymphocyte count, integrates the three key variables identified in our model (CRP, NE, and lymphocyte count) and provides a precise quantification of the systemic inflammatory burden in cancer patients. A large multicenter study (*N* = 6,359) demonstrated that patients with high IBI exhibited significantly lower 5-year survival rates compared with those with low IBI (45.7% vs. 69.1%, HR = 1.103 per SD, *p* < 0.001), and that IBI outperformed traditional inflammatory markers in prognostic prediction, with an improvement in AUC of 15%–20% (26). Similarly, Diem et al. reported that in patients with metastatic non-small cell lung cancer (NSCLC), a high pretreatment neutrophil-to-lymphocyte ratio (NLR) was associated with significantly reduced overall survival (HR = 3.64, *p* < 0.001) (27). These findings are highly consistent with the present study, in which patients in the deterioration group already exhibited markedly elevated CRP and neutrophil levels (OR = 1.005 and 1.086, respectively), along with pronounced lymphocyte depletion (OR = 0.325) at the time of admission. Collectively, these results suggest that a state of “high inflammation–low immunity” imbalance may be closely associated with acute clinical deterioration in lung cancer patients.

From a practical perspective, the five predictors in this model—ADL score, lymphocyte count, CRP, respiratory rate, and neutrophil count—can be obtained through bedside nursing assessment and routine laboratory testing, including complete blood count and arterial blood gas analysis, which are typically available within 30 min via urgent or point-of-care testing. This combination of simple clinical indicators may assist in risk stratification at the time of assessment, helping to identify patients who may benefit from intensified surveillance while avoiding unnecessary escalation of care in low-risk individuals. A positive prediction would prompt intensified nursing monitoring, repeated clinical reassessment, and early multidisciplinary consultation rather than immediate admission to intensive care. However, these potential clinical applications require prospective validation. Nevertheless, this study has several limitations. First, the model was developed and validated in a single-center retrospective cohort, and the internal validation set contained only 36 deterioration events; both factors limit the precision of the performance estimates and the generalizability of the model. Multicenter external validation is therefore still needed, and the findings should be regarded as preliminary. Second, as a retrospective analysis, the study is inevitably subject to a certain degree of selection bias and missing data. In particular, the 215 patients excluded because of missing ADL data could not be compared with the included cohort, and this exclusion may have introduced selection bias. In addition, the model primarily relies on baseline data at the time of admission and does not fully incorporate dynamically changing laboratory parameters, which may limit its ability to capture the temporal evolution of clinical deterioration. Third, although the five-variable SVM model achieved higher discrimination than NEWS2 and MEWS in the validation set (AUC: 0.816 vs. 0.639 and 0.609), the discrimination of the five-variable model was almost identical to that of the ADL score alone (AUC: 0.816 vs. 0.815); the added inflammatory and physiologic variables therefore contributed little extra discriminative value, and whether the more complex model adds meaningful clinical benefit over this simple measure still needs confirmation in external cohorts. Fourth, NEWS2 and MEWS were used only as comparators and were not entered into the feature selection procedure. Because supplemental oxygen use and level of consciousness were not recorded in the electronic records, these two components were not included in the scores, which may have produced slightly conservative values for both tools. Fifth, cancer stage, ECOG performance status, and treatment modality were not available, so these potential confounders could not be adjusted for. Because such variables relate to both functional status and systemic inflammation, the strong contribution of the ADL score may partly reflect unmeasured disease stage and performance status, and model performance could differ once these data become available. Finally, future studies may consider integrating genomic sequencing data or tumor microenvironment biomarkers to construct multi-omics models.

## 5 Conclusion

This study developed and internally evaluated an SVM model using readily accessible clinical indicators to predict clinical deterioration in hospitalized lung cancer patients. The model demonstrated satisfactory discriminative ability and calibration in internal validation. SHAP analysis identified ADL score, CRP, and neutrophil count as key predictors, enhancing model transparency. These findings suggest that the model may help to identify patients at high risk of deterioration at admission. Its performance, however, given the single-center retrospective design with few events in the internal validation set, the results should be regarded as preliminary. External and ideally prospective validation is needed before clinical implementation.

## Data Availability

The raw data supporting the conclusions of this article will be made available by the authors, without undue reservation.

## Reporting guidelines

This study rigorously followed the TRIPOD-AI guideline (Transparent Reporting of a multivariable prediction model for Individual Prognosis Or Diagnosis—AI extension) (28) to ensure full compliance with its checklist during model development, validation, performance evaluation, and reporting.
